# Tritium-Labeled Compounds IX. Determination of Isotope Effects in Reactions Yielding Water-*t* from Nonvolatile Reactants. Oxidation of Aldoses-*1*-*t* With Iodine

**DOI:** 10.6028/jres.067A.054

**Published:** 1963-12-01

**Authors:** Horace S. Isbell, Lorna T. Sniegoski

## Abstract

A convenient method is presented for estimating isotope effects in reactions which yield water-*t*. The water-*t* is separated by freeze-drying, and k*/k is calculated from the extent of the reaction and the ratio of the radioactivity of the water-*t* to that of the initial reactant. Application of the method to the oxidation of aldoses-1-*t* at 25 °C with iodine in alkaline solution gives values for k*/k ranging from 0.12 to 0.17, with an average of 0.14. There are no significant differences in the values of k*/k obtained for aldoses differing in configuration, or for pentoses and hexoses. The isotope effect corresponds to a ratio of one to seven for the respective rates of reaction of the labeled and unlabeled molecules.

## 1. Discussion

### 1.1. Determination of Isotope Effects

Isotope effects, or differences in the rates of reaction for isotopically labeled and unlabeled molecules, provide a tool for study of reaction mechanisms [1].^1^ The isotope effect, k*/k, can be determined by various techniques, some of which have been described in prior publications from this laboratory [2, 3].

A simple method has now been developed for determining k*/k for reactions in which water-*t* is formed from a nonvolatile reactant. The method is based on the separation and analysis of the water-*t* formed in the reaction. The ratio of k*/k is then calculated from the equation of Stevens and Attree [4] as modified by Ropp [5]:

k*/k=log(1−rf)/log(1−f) (1)

In this equation, *f* is the fraction of initial reactant that has been consumed, and *r* is the ratio of the molar specific radioactivity of the accumulated product (of partial reaction) to that of the initial reactant.

In the experimental procedure developed, the reaction is allowed to proceed to the desired extent. The fraction *f* is estimated from the amount of the reagent used or by other convenient means. The solution containing the products of partial reaction and the residual reactant is diluted with water to a known volume. A portion of this solution is freeze-dried, the water-*t* obtained by sublimation is assayed, and the total activity of the water-*t* (the product of partial reaction) is calculated. The total activity of the initial reactant is obtained from the specific activity and weight of the aldose-*1*-*t* The ratio of the activity of the water-*t* to the activity of the initial reactant equals *rf*. Substituting the values of *f* and *rf* in eq (1) gives k*/k.

The method can be applied to a wide variety of reactions which yield water-*t* from nonvolatile reactants; it is illustrated here by a study of the oxidation of aldoses-*1-t* with iodine in alkaline solution.

### 1.2 Oxidation of Aldoses-*1-t* With Iodine in Alkaline Solution

In a prior investigation, Isbell, Sniegoski, and Frush [2] measured the isotope effect for the oxidation of d-glucos*e-1-t* by iodine in alkaline solution by means of their double-label technique. The observed value of k*/k (0.17) is substantially the same as that (0.15) previously found by Kaplan [6] for the bromine oxidation of ethanol-*1*-*t*, but differs greatly from that (0.53) found for the bromine oxidation of *α*-d-glucose-*1-t* [7]. The rate of oxidation of *α*-d-glucose-*1*-*t* with bromine in slightly acid solution depends, in part, on the rate of anomerization to the rapidly oxidized *β*-d-glucose-*1*-*t* [8]. The anomerization reaction has a weak, secondary isotope-effect, whereas the direct oxidation has a strong, primary isotope-effect. In the oxidation of aldoses with iodine in alkaline solution, equilibrium is maintained throughout the reaction and, consequently, the isotope effect is not influenced by a rate-controlling anomerization. In view of this fact, it seems probable that the value of k*/k for the reaction is typical of a reaction involving rupture of the C-1 to H bond of an aldose-*1-t* present in a steady- state equilibrium. The value of k*/k for this type of reaction is needed for interpreting many of the reactions of labeled carbohydrates.

To ascertain whether all aldoses, on oxidation with iodine, give essentially the same value of k*/k, a series of measurements was made with aldoses-*1*-*t* of diverse types. The results, summarized in table 1, show that the values of k*/k are essentially constant and that there are no significant differences in the values for aldoses differing in configuration, or for pentoses and hexoses. The average value of k*/k (0.14) and the lack of its dependence on the structure of the aldose are in accord with the conclusion that the C-1 to H bond is ruptured in the rate-determining step.

In addition to the measurements of k*/k given in table 1, measurements were also made for d-mannose-*1-t* (table 2) by means of the double-label technique previously developed [2]. The results obtained by the two methods are in good agreement, considering that the values for the water-*t* method are based on the early part of the reaction period, whereas those for the double-label method are based on the latter part.

## 2. Experimental Procedure

### 2.1. Materials and Apparatus

#### a. Labeled Aldoses

The sugars labeled at C-1 with tritium were prepared by reducing the corresponding lactones with LiBH_4_-*t* [9]. The purity of each sugar was checked, before use, by paper chromatography. One chromatogram was developed with 1-butanol—acetic acid—water (4:1:5, upper phase), and another with 2-butanone—acetic acid—water saturated with boric acid (9:1:1). The chromatograms were scanned with a gas-flow, windowless counter, and then sprayed with aniline hydrogen phthalate. Whenever either chromatogram showed the presence of more than one substance, the labeled aldose was recrystallized and then rechromatographed. The pure, labeled sugars, generally having an activity of about 1 *μc*/mg, were assayed with a liquid scintillation counter.

d-Mannose-*2*-*C*^14^ was prepared by the procedure given in [10], and d-mannose-*2*-*C*^14^-*1*-*t* by recrystallizing a suitable mixture of d-mannose-*2*-*C*^14^ and d-mannos*e-1-t* from water—methanol—2-propanol. The activity of the doubly labeled d-mannose, determined as described in sec. 2.1.c was about 1 *μ*c/mg for tritium and 0.1 *μ*c/mg for carbon-14.

#### b. Standard Solutions

0.04 *N* Iodine (containing 4 g of potassium iodide per 100 ml of solution) and 0.04 *N* sodium hydroxide were used.

#### c. Counting Equipment

The counting was done with a Packard Tri-Carb Liquid Scintillation Spectrometer, Series 314E [11]. The counter was operated at 1325 v, with discriminators so adjusted that one scaler (red) counted at 8 to 40 v and the other (green) at 10 to 100 v; the temperature of the counting chamber was +5 °C. The scintillation solution [12] contained, per liter, 7 g of PPO (2,5-diphenyloxazole, scintillation grade), 0.3 g of dimethyl POPOP [2,2′-*p*-phenylenebis(4- methyl-5-phenyloxazole), scintillation grade], and 100 g of naphthalene, in dioxane (Eastman). Aqueous solutions (100 *μ*l, generally containing about 0.02 *μ*c of tritium) were counted in 10 ml of the scintillation solution. In each instance, duplicate samples were prepared and counted to a total of 10,000 counts, to give a statistical accuracy of ± 1 percent.

### 2.2 Determination of Tritium

The counting efficiency for tritium was determined as described in sec. 2.1.c, by analysis of a standard sample of tritiated water. A factor, expressed as *μ*c of tritium per count per sec, was calculated from the count (red scaler) and the known activity. Samples containing unknown amounts of tritiated water were analyzed in the same way as the standard, and the activity was calculated by means of the factor.

### 2.3. Determination of Carbon-14 and Tritium in the Same Sample

The determination of carbon-14 and tritium in the same sample was carried out with the scintillation counter so adjusted that each isotope was counted efficiently in one scaler and inefficiently in the other [11]. The efficiencies for carbon-14 and for tritium with each scaler were determined by separately counting standard samples of d-glucose-*1*-*C*^14^ and d-glucose-*1*-*t*. The activities of unkown samples were then calculated by the following equations:

$$A^{T}=[R_{r}-R_{g}(E_{r}^{C}/E_{g}^{C})]/[E_{r}^{T}-E_{g}^{T}(E_{r}^{C}/E_{g}^{C})]=k_{1}R_{r}-k_{2}R_{g}$$ (2)

$$A^{C}=[R_{r}-R_{g}(E_{r}^{T}/E_{g}^{T})]/[E_{r}^{C}-E_{g}^{C}(E_{r}^{T}/E_{g}^{T})]=k_{3}R_{r}-k_{4}R_{g}$$ (3)

where *A^T^*=disintegrations per second due to tritium, *A^c^*=disintegrations per second due to carbon-14, *R_r_*=count rate on the red scaler, *R_g_*=count rate on the green scaler, $E_{r}^{C}$= counting efficiency of carbon-14 on the red scaler, $E_{g}^{C}$= counting efficiency of carbon- 14 on the green scaler, $E_{r}^{T}$= counting efficiency of tritium on the red scaler, and $E_{g}^{T}$= counting efficiency of tritium on the green scaler. The combined constants, *k*_1_, *k*_2_, *k*_3_ and *k*_4_, were derived from the experimentally determined counting-efficiencies. Under the conditions described in sec. 2.1.C, a mixture containing carbon-14 and tritium gave values of *0.126*, *0.707*, 0.175, and 0.082 for $E_{r}^{C}$, $E_{g}^{C}$, $E_{r}^{T}$ and $E_{g}^{T}$, respectively.

### 2.4. Determination of k^*^/k by the Water-*t* Method

The procedure for studying the oxidation of aldoses-*1*-*t* was as follows: Several weighed samples (approximately 10 mg each) of each aldose-*1*-*t* were oxidized at room temperature by dissolving each in 0.5 ml of water, adding sufficient 0.04 *N* iodine to give a small degree of oxidation (about 10, 20, or 30%), and then adding dropwise 1.5 equivalents of 0.04 *N* sodium hydroxide. After 15 min, each solution was transferred to a 10-ml volumetric flask and diluted to volume. Then, about 2 ml of the solution was transferred to a standard-tapered male-joint, round-bottomed flask containing about 50 mg of sodium carbonate. The solution was frozen, and the ice-*t* was sublimed under vacuum into another flask. The sublimate was warmed to room temperature, and 100-*μ*l samples of the liquid were removed and assayed with the liquid scintillation counter by the procedure given in sec. 2.1.c. In each instance, the amount of tritium in the reaction mixture was calculated from the amount of the aldose-*1*-*t* added; the amount of water-*t* formed was calculated from the volume of the reaction mixture and the specific activity of the sublimed water. The extent of the reaction, *f*, was calculated from the amount of iodine added.

In a typical measurement, a 9.241-mg sample of d-glucose-*1*-*t* containing 7.43 *μ*c of tritium was allowed to react with 0.51 ml of 0.04 *N* iodine and 0.77 ml of 0.04 *N* sodium hydroxide. The reaction mixture was diluted to 10 ml with water, and a 2-ml sample was freeze-dried. A 100-*μ*l sample of the water-*t*, obtained by freeze-drying, assayed 15.36 counts per second, corresponding to 0.00252 *μ*c in the sample or 0.252 *μ*c in 10 ml. Thus, *rf*= 0.252/7.43 = 0.0339. The amount of iodine added corresponded to *f*=0.20. Substitution of these values in eq (1) gives 0.15 for k*/k.

### 2.5. Determinatian of k*/k by Double-Labeling

A weighed sample (10 mg) of the C^14^-labeled aldose-*1*-*t* was dissolved in water (0.5 ml) and treated with sufficient 0.04 *N* iodine and 0.04 *N* sodium hydroxide to oxidize between 75 and 90 percent of the aldose. After 15 min, the solution was diluted with 5 ml of water, and 100 mg of the unlabeled aldose was added as carrier. The solution was passed through 20 ml of mixed cation- and anion-exchange resins (Amberlite IR-120 (H^+^) and Duolite A4). The effluent was concentrated under reduced pressure to a sirup from which the labeled aldose was crystallized; it was recrystallized twice from methanol— 2-propanol. After being dried, the purified carrier- mixture was assayed for carbon-14 and tritium by the procedure given in section 2.3. A sample of the original C^14^-labeled aldose-*1*-*t* was also assayed. The amount of the residual reactant was calculated from the equation:

$$w=\frac{s^{\prime }w^{\prime }}{s-s^{\prime }}$$ (4)

where *w* is the weight of the residual reactant, *w*′ is the weight of the nonradioactive carrier added, and *s* and *s*′ are the specific activities (in carbon-14) of the initial reactant and the carrier mixture, respectively. The fraction of aldose oxidized, *f*, is the ratio of reactant consumed to initial reactant.

The value of k*/k was calculated as previously described [2] from the equation:

k*/k=1+[log(p/p°)/log(1−f)] (5)

where *p°* is the ratio of tritium to carbon-14 in the initial reactant, and *p* is the ratio in the residual reactant. The results obtained for d-glucose-*6*-*C*^14^-*1-t* and d-mannose-*2*-*C*^14^-*1*-*t* are givin in table 2.

## Figures and Tables

**Table 1:** Isotope effects in oxidation of aldoses-1-*t* with iodine in alkaline solution at 25 °C

Aldose-*1*-*t*	Fraction of aldose oxidized	Radioactivity in original reactant	Radioactivity of water-*t* formed	Isotope effect
				
	*f*	*μc*	*μc*	k*/k
l-Arabinose	0.100	6.05	0.092	0.15
	.200	6.30	.194	.14
	.300	5.64	.262	.13
				Avg. 0.14
d-Xylosc	.165	15.90	.317	.12
	.200	13.78	.511	.17
	.303	13.83	.756	.16
				Avg. 0.15
d-Glucosc	.100	7.60	.120	.15
	.200	7.43	.252	.15
	.300	7.56	.409	.16
				Avg. 0.15
d-Mannose	.107	9.82	.143	.13
	.198	7.55	.214	.13
	.313	7.47	.334	.12
				Avg. 0.13
d-Galactose	.101	6.26	.082	.12
	.199	6.35	.186	.13
	.317	6.42	.340	.14
				Avg. 0.13
				
d-Talose	.100	10.86	.194	.17
	.200	11.26	.340	.14
	.300	10.09	.515	.15
				Avg. 0.15
l-Rhamnose monohydrate	.100	16.71	.239	.14
	.200	11.28	.330	.13
	.300	12.40	.504	.12
				Avg. 0.13

**Table 2:** Determination of isotope effects in the oxidation of aldoses-1-*t* with iodine in alkaline solution by the double- label technique [2]

Fraction of aldose oxidized	Ratio of functional to reference isotope in carrier mixture	Isotope effect

d-Glucose-*6*-*C*^14^-*1*-*t*^a^		
*f*	*p*	k*/k
0.0	9.12	
.761	30.89	0.15
.785	30.66	.21
.839	43.05	.15
.882	52.17	.18
		Avg. 0.17

d-Mannose-*2*-*C*^14^-*1-t*		
0.0	7.72	
.726	23.43	.14
.756	26. 39	.13
		Avg. 0.14

a These measurements for d-glucose- *6-C*^14^-*1-t* were previously reported in [2].

## Footnotes

1 Figures in brackets indicate the literature references at the end of this paper.
